# Specificity in clustering of gene-specific transcription factors is encoded in the genome

**DOI:** 10.1093/nar/gkaf625

**Published:** 2025-07-12

**Authors:** Shivali Dongre, Nadine L Vastenhouw

**Affiliations:** Center for Integrative Genomics, University of Lausanne, Quartier Sorge, 1015 Lausanne, Switzerland; Center for Integrative Genomics, University of Lausanne, Quartier Sorge, 1015 Lausanne, Switzerland

## Abstract

Gene-specific transcription factors (TFs) often form clusters in the nucleus. Such clusters can facilitate transcription, but it remains unclear how they form. It has been suggested that clusters are seeded by the sequence-specific binding of TFs to DNA and grow by interactions between intrinsically disordered regions (IDRs) that bring in more TFs. In this model, specificity in TF clustering must be provided by the IDRs. To investigate this model, we studied TF clustering by quantitative imaging of Nanog, Pou5f3, and Sox19b in zebrafish embryos. Using mutant TFs, we show that the formation of a TF cluster requires the DNA-binding domain (DBD) as well as at least one of its IDRs. Importantly, IDRs are not sufficient to join a pre-existing cluster. Rather, both IDR and DBD are needed. Finally, using chimeric TFs, we show that while IDRs are required to join a cluster, they are quite promiscuous, and it is the DBD that provides specificity to the clustering of a TF. Thus, for any TF to join a cluster, motif recognition is required, which explains the specificity in TF cluster formation. Taken together, our work provides an alternative model for how specificity is achieved in the organization of transcriptional machinery in the nucleus.

## Graphical Abstract

**Graphical Abstract:**
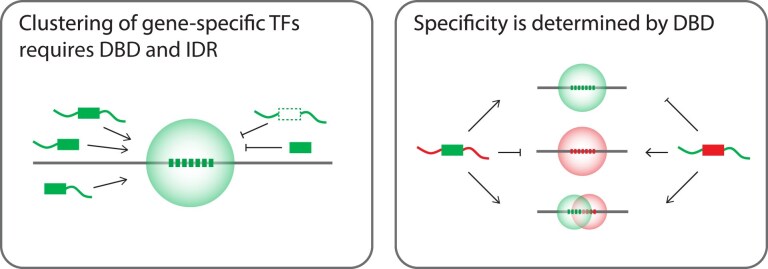

## Introduction

Transcription is a fundamental biological process. To transcribe a gene in the right place and at the right time, several factors need to act in a coordinated manner. Gene-specific transcription factors (TFs) recognize and bind to specific DNA sequences within promoter or enhancer regions and then recruit transcriptional co-activators and general TFs to activate transcription. Gene-specific TFs, cofactors, general TFs, and RNA Polymerase II are often concentrated in the nucleus in what have been called hubs, clusters, condensates, or transcription bodies [1–9]. These clusters are often found at regulatory elements like enhancers [3, 10, 11]. It has been proposed that the local increase in protein concentration in clusters accelerates biochemical reactions [12], and in line with this, clustering of transcriptional machinery at regulatory elements has been correlated with transcriptional activity [13–17].

Gene-specific TFs are generally composed of distinct functional domains, such as a DNA-binding domain (DBD, e.g. homeodomain, zinc finger domain) and a transactivation domain. The DBD is generally well-structured, allowing for specific recognition and binding to target DNA sequences [18]. Transactivation domains, in contrast, often lack a fixed 3D conformation and are part of the intrinsically disordered regions (IDRs) of TFs [19, 20]. The disordered nature of transactivation domains allows TFs to interact with a variety of proteins like co-activators, chromatin remodelers, and components of the general transcription machinery. IDRs often extend beyond the transactivation domain and can serve various functions beyond transcriptional activation, such as molecular signaling or serving as scaffolds for protein complexes [18]. The structural flexibility of IDRs is crucial for the dynamic and versatile nature of transcriptional regulation, as it enables TFs to form transient and multivalent interactions. In fact, a prevalent model for the clustering of gene-specific TFs postulates that TFs bind to DNA with their DBD and recruit additional molecules into the cluster by IDR–IDR interactions.

The clustering of transcriptional machinery has been best studied for the general transcriptional machinery. Here, there is evidence for IDRs being required and sufficient to recruit proteins into a condensate [3, 11, 21–23]. Specificity, in this case, is provided by the IDRs itself [3, 21, 22]. There is also a gene-specific TF (ß-catenin, with TCF/LEF) for which the IDR has been shown to be sufficient to recruit the protein into a MED1 condensate [4]. Moreover, IDRs have been shown to contribute to TF binding specificity *in vivo* [24]. And finally, we know that DNA-binding TFs form clusters in which the TF molecules are highly dynamic and do not all bind to DNA at any given time [18, 23, 25, 26]. Together this has led to the model that an IDR may be sufficient to recruit a TF into a cluster and provide the specificity needed to create distinct clusters.

To investigate this model for gene-specific TFs, we tested the role of IDRs and DBD in the clustering of Nanog, Pou5f3, and Sox19b in zebrafish embryos. These three gene-specific TFs are required to activate transcription in the zebrafish embryo, and we and others have previously shown that they form clusters in the nucleus [26–30]. The availability of mutant embryos that lack these TFs, as well as the ease of supplementing these embryos with synthetic messenger RNAs (mRNAs) coding for mutant forms of the same TFs, make the zebrafish embryo an excellent model to study how the different domains of TFs contribute to cluster formation. Using quantitative live imaging, we find that Nanog-, Pou5f3-, and Sox19b-clusters in the nucleus are DNA-seeded. The number of clusters scales with the amount of TF and high affinity sites form clusters first. Our structure-function analysis revealed that the DBD and at least one IDR are required to form a cluster, which is in line with the existing model. Remarkably, however, we find that to join a pre-existing cluster, IDRs are not sufficient, and the DBD is also required. Finally, using chimeric proteins, we show that IDRs are quite promiscuous, and it is the DBD that confers specificity to the IDR. Taken together, our results suggest a model where the specificity in the clustering of gene-specific TFs is determined by their DBD and thus encoded in the genome.

## Materials and methods

### Zebrafish husbandry and manipulation

Zebrafish were maintained and raised under standard conditions and according to Swiss regulations (canton Vaud, license number VD-H28). Wild-type (TLAB) and mutant (*nanog^−/−^*, *sox19b^−/−^*, and *pou5f3^−/−^*) fish were used for this study. The *nanog^−/−^* mutant has a 10 bp deletion in the first exon, leading to a frameshift mutation and a truncated protein [28]. In the *pou5f3^−/−^* and *sox19b^−/−^* mutants, the entire coding region was deleted as described [31]. Embryos were collected immediately after fertilization. The embryos were grown at 28°C, and the developmental stage was determined as described by Kimmel *et al.* [32]. Developmental stages of mutant and injected embryos were determined by comparing them to wild-type embryos obtained at the same time and grown under identical conditions.

### Genotyping mutant fish

Genotypes of maternal zygotic (MZ) fish (*nanog^−/−^*, *sox19b^−/−^*, and *pou5f3^−/−^*) were confirmed by phenotyping and/or genotyping. For genotyping, adult fish were fin-clipped and the genomic DNA was extracted according to the protocol described by Meeker and colleagues [33]. Genotyping polymerase chain reactions (PCRs) for the identification of Nanog, Sox19b, and Pou5f3 mutants were performed as described previously [28, 34].

### Generating tetraploid embryos

Tetraploid embryos were generated using the HSII protocol [35] with some minor changes. Briefly, two water baths were maintained at 28°C and 42.2°C. Beakers containing embryo medium (0.3× Danieau’s) were placed in each water bath well in advance of embryo collection to allow the medium to equilibrate to the respective temperatures. Embryos were collected immediately after fertilization and transferred to a tea strainer, which was immersed in the embryo medium at 28°C. At 22 min post fertilization, the tea strainer containing the embryos was transferred to the embryo medium at 42.2°C for a heat shock for 2 min. Immediately after the heat shock, the embryos were transferred to a 28°C incubator to recover. After 10 min of recovery, while the embryos were still at the one-cell stage, they were microinjected as described earlier. Tetraploids were identified based on two parameters: (i) a delay of one cell cycle in the tetraploid embryos when compared to embryos that were not subjected to heat shock [35] and (ii) counting the number of *mir430* transcription bodies, labeled with MiR430 MoVIE [36].

### Microinjection of RNA and morpholinos

To visualize TFs (full-length, mutated, and chimeric), embryos were injected at the one-cell stage with the mRNA coding for a specific TF-fluorophore fusion. The fusion mRNA was translated within the embryo, and the resulting protein visualized by spinning disk microscopy. The *in vitro* synthesized mRNA for the TF-fluorophore fusion constructs was injected at the concentration and molar amount that is indicated in the table below. We ensured that the molar amount of Nanog, Pou5f3, and Sox19b deletion constructs, as well as the SNS, FNF, and NSN chimeras, was equivalent to the full-length construct containing the corresponding DBD. To label nascent MiR430 RNA, Lissamine-labeled anti-MiR430 morpholino (5′-TCTACCCCAACTTGATAGCACTTTC-3′-Lissamine, Gene Tools) was injected in one-cell stage embryos at 14 fmole/embryo as described previously [36].

## Amount of RNA injected for each of the constructs

See Table 1.

**Table 1.** Amount of RNA injected for each of the constructs

Construct	RNA length	Amount injected	Number of moles	No. of RNA molecules
Nanog FL-mNeonGreen	1902 nt	180 pg	295.3 amol	1.68 × 10^8^
Nanog HD Only-mNeonGreen	930 nt	88 pg	295.2 amol	1.68 × 10^8^
Nanog ΔHD-mNeonGreen	1701 nt	161 pg	295.3 amol	1.68 × 10^8^
NanogΔN-mNeonGreen	1560 nt	147 pg	294.0 amol	1.67 × 10^8^
NanogΔC-mNeonGreen	1317 nt	125 pg	296.2 amol	1.68 × 10^8^
Sox19b FL-mScarlet-I	1614 nt	150 pg	290.0 amol	1.65 × 10^8^
Sox19b HMG Domain only- mScarlet-I	984 nt	92 pg	291.7 amol	1.66 × 10^8^
Sox19b ΔHMG-mScarlet-I	1416 nt	132 pg	290.9 amol	1.65 × 10^8^
Sox19bΔN-mScarlet-I	1452 nt	135 pg	290.1 amol	1.65 × 10^8^
Sox19bΔC- mScarlet-I	1128 nt	105 pg	290.4 amol	1.65 × 10^8^
Pou5f3 FL-mNeonGreen	2229 nt	150 pg	210.0 amol	1.19 × 10^8^
Pou5f3 DBD Only-mNeonGreen	1272 nt	86 pg	211.0 amol	1.20 × 10^8^
Pou5f3ΔDBD-mNeonGreen	1767 nt	120 pg	211.9 amol	1.20 × 10^8^
Pou5f3ΔN-mNeonGreen	1488 nt	100 pg	209.7 amol	1.19 × 10^8^
Pou5f3ΔC-mNeonGreen	2019 nt	136 pg	210.2 amol	1.19 × 10^8^
NSN-mNeonGreen	1923 nt	180 pg	292.1 amol	1.66 × 10^8^
SNS-mNeonGreen	1641 nt	155 pg	294.7 amol	1.67 × 10^8^
FNF-mNeonGreen	2361 nt	220 pg	290.8 amol	1.65 × 10^8^

### Classification of disordered regions

To classify regions of proteins as structured or intrinsically disordered, we followed criteria as described before [37]. Disorder propensity was predicted using OdinPred [38], which assigns scores from 0 to 1, where values ≥0.5 indicate a propensity for disorder, and values <0.5 indicate a propensity for order. We applied a minimum threshold of 30 consecutive ordered residues to define a structured domain. Regions shorter than this threshold, even if showing partial order, were not considered independently structured. Based on these criteria, the DBDs and IDRs of Nanog, Sox19b, and Pou5f3 were predicted as shown in Supplementary Fig. S2 and Fig. 3.

### Generation of deletion constructs and chimeras

Full-length plasmids for Nanog, Pou5f3, and Sox19b in the pCS2+ vector, tagged with mNeonGreen or mScarlet-I at the C-terminus, were obtained from prior work [26]. Deletion constructs for Nanog were previously established [26], while those for Pou5f3 and Sox19b were generated using the plasmids with the sequence encoding the full-length proteins as templates. All around PCR was performed on the full-length constructs in pCS2+ by using primers flanking the segments that were to be deleted. The exact number of amino acids that were deleted is mentioned in Fig. 3A. To make the chimeras, the individual segments were amplified by PCR and cloned using the Gibson Assembly kit following the manufacturer’s instructions. The SNS chimera was generated by fusing the N-terminal part of Sox19b (aa 1-53), Nanog HD (aa 195-254), and the C-terminal part of Sox19b (aa 113-284). The FNF chimera was generated by fusing the N-terminal part of Fus (aa 1-299), Nanog HD (aa 195-254), and the C- terminal part of Fus (aa 359-514). The NSN chimera was generated by fusing the N-terminal part of Nanog (aa 1-194), the Sox19b HMG domain (aa 195-262), and the C-terminal part of Nanog (aa 263-392). A list of all primers used for cloning these constructs can be found below. All positive clones were validated by sequencing.

## List of primers used for cloning

See Table 2.

**Table 2.** List of primers used for cloning

Primer name	Sequence
NSN-backbone_gibson_FWD	AGG AGT ACC CCG ACT ACA AAA GAG ACA GCA GTT GGA TGA CTG AGA
NSN-backbone_gibson_REV	GCG TTC ATG GGC CGC TTG ACC AGG GTC GGA GGC CGG ACA G
NSN-insert_gibson_FWD	CTG TCC GGC CTC CGA CCC TGG TCA AGC GGC CCATGA ACG C
NSN-insert_gibson_REV	GTC ATC CAA CTG CTG TCT CTT TTG TAG TCG GGG TAC TCC TTC A
SNS-backbone_gibson_FWD	TGA AAC TCA AGA GGC ATC AGT ATA AGC CCC GGC GCA AGA C
SNS-backbone_gibson REV	GCA GCC CGG GTC TTG CGG GGT TTG TCC ATC GGG TCC ACG C
SNS-insert_gibson_FWD	GCG TGG ACC CGA TGG ACA AAC CCC GCA AGA CCC GGG CTG C
SNS-insert_gibson_REV	GTC TTG CGC CGG GGC TTA TAC TGA TGC CTC TTG AGT TTC ATC CTG CGG
Fus_daniorerio_FWD	CAT GAG GCC GGC CAT GGC GTC AAA TGA TTA TGG
Fus_daniorerio_REV	CAT GAG GCG CGC CGT AAG GGC GGT CTC
FNF-backbone_gibson_ FWD	TGA AAC TCA AGA GGC ATC AGA GAG CTG AGT TTG GTC GTG G
FNF-insert_gibson_FWD	AGC AGG ATA ACT CTG ACA ACC CCC GCA AGA CCC GGG CTG C
FNF-insert_gibson_ REV	CCA CGA CCA AAC TCA GCT CTC TGA TGC CTC TTG AGT TTC ATC CTG CGG
Pou5f3_DeltaN_FWD	GAG GAG ACT CTG ACT ACT GAA G
Pou5f3_DeltaN_REV	CAT GAA TTC GAA TCG ATG GGA TCC
Pou5f3_DeltaDBD_FWD	TTG CCC TTT GAT GAC GAG TG
Pou5f3_DeltaDBD_REV	TTC CTC AGA ATC ACT GCA TCC
Pou5f3_DeltaC_FWD	AGC TAG ACG CTT TCC CTT CTG TC
Pou5f3_DeltaC_REV	AGC TAG ACG CTT TCC CTT CTG TC
Pou_DBD ONLY_FWD	CAT GAG GCC GGC CAT GGA GGA GAC TCT GAC TAC TGA AG
pou5f3_DBD ONLY_REV	CAT GAG GCG CGC CAG CTA GAC GCT TTC CCT TCT G
Sox_HMG Only_FWD	CAT GAG GCC GGC CAT GGT CAA GCG GCC
Sox_HMG Only_REV	CAT GAG GCG CGC CAG CTT TGG TCT TGC G
Sox_DeltaN_FWD	GTC AAG CGG CCC ATG AAC
Sox_DeltaN_REV	CAT CAT GAA TTC GAA TCG ATG GGA
Sox_DeltaHMG_FWD	TAT AAG CCC CGG CGC AAG
Sox_DeltaHMG_REV	CCG CTT GAC TTT GTC CAT
Sox_DeltaC_FWD	ACT CAC ATC ACC GGG GGA GGA ATG
Sox_DeltaC_REV	GAG AGC TTT GGT CTT GCG CCG

### mRNA production

Prior to *in vitro* transcription, 5–10 μg of plasmid was linearized with the restriction enzyme NotI (NEB, R3189L). The linearized plasmids were run on a gel to ensure complete digestion and purified using a PCR and gel purification kit, following manufacturer’s instructions (Qiagen, 28506). The SP6 mMessage mMachine *in vitro* transcription kit (Thermo Fisher Scientific, AM1340) was used for *in vitro* transcription, according to manufacturer’s instructions. One microliter of the mRNA was run on an agarose gel to check if a band of the correct size was obtained. The RNA was purified using Qiagen’s RNeasy MinElute kit, following manufacturer’s instructions (Qiagen, 74204). The purified product was diluted to a concentration of 600 ng/μl, aliquoted as 1 μl aliquots, and stored at −70°C for several months.

### Western blot

Embryos were collected at sphere stage, manually deyolked, and immediately flash-frozen in liquid nitrogen. A total of 10 embryos per condition were collected. For protein extraction, embryo lysates were thawed on ice, and 2× sodium dodecyl sulfate loading buffer was added. Samples were briefly vortexed, incubated on ice for 5 min, vortexed again, and then heated at 95°C for 5 min. Lysates were briefly centrifuged before gel loading. Samples were resolved on mPAGE® 4-20% Bis-Tris Precast Gels (MP42G12, Millipore) using 1× MOPS (3-(N-morpholino)propanesulfonic acid) running buffer. Electrophoresis was performed at 120 V for 1 h or until the dye front reached the bottom of the gel. Protein transfer was carried out using the Bio-Rad Trans-Blot Turbo semi-dry transfer system onto a nitrocellulose membrane. Post-transfer, membranes were stained with Ponceau to confirm efficient transfer, then rinsed to remove the stain. Membranes were blocked in 5% nonfat dry milk prepared in Phosphate Buffered Saline with Tween-20 (PBST) (1× PBS with 0.1% Tween-20) for 1 h at room temperature (RT) on a rotor. Primary antibodies were diluted in 5% milk and incubated overnight at 4°C with shaking. Following incubation, membranes were washed three times for 10 min each in PBST. Secondary antibodies, also diluted in 5% milk, were then applied for 1 h at RT with shaking, followed by three 10-min washes in PBST. Membranes were blotted dry on a paper towel, incubated with ECL™ Western Blotting Reagents (Amersham, RPN2106), and imaged using a Vilber Lourmat Fusion FX imaging system.

## Antibody details

See Table 3.

**Table 3.** Antibody details

Antibody	Host species	Dilution	Supplier (catalogue no.)
Anti-HA	Mouse	1:2000	Santa Cruz Biotechnology (sc-7392)
Anti-HA-HRP	Rat	1:2000	Roche (12013819001)
Anti-mNeonGreen	Rabbit	1:2000	Cell Signaling Technology (E8E3V, #55074)
Anti-Mouse IgG (H + L), HRP conjugate	Goat	1:5000	Promega (W4021)
Anti-Rabbit IgG (H + L), HRP conjugate	Goat	1:5000	Promega (W4011)

### Electrophoretic mobility shift assay

DNA-binding domains of Nanog and Pou5f3, fused to mNeonGreen, were expressed in *Escherichia coli* with a C-terminal histidine tag and purified using Ni-NTA agarose. For Sox19b, two versions of the HMG domain (a truncated 68 amino acid version and a longer 79 amino acid version) were generated and fused to mScarlet-I. Probes [GGGGCTGGAGGTGTTGATGGCTGTTT (for Nanog), GGGCGCCTGTATGCTAATACAGTCTT (for Pou5f3), and CTGAAAGAAAACTCTTTGTTTGGATGCTAATGGGATACTAAGCTGA (for Sox19b)] [39, 40] were biotinylated using the Pierce™ Biotin 3′ End DNA Labeling Kit (Thermo Scientific, 89818) according to the manufacturer’s instructions. Binding reactions were performed using the LightShift™ Chemiluminescent EMSA Kit (Thermo Scientific, 20148), following the kit protocol. Reactions were resolved on a 4%, 29:1 acrylamide:bis-acrylamide gel containing 5% glycerol in 0.5× TBE buffer. Gels were pre-run for 1 h at 100 V, and the binding reactions were then run at 100 V until the dye front reached the bottom. For transfer, a wet transfer protocol was used with a positively charged nylon membrane. Transfer was carried out for 45 min at 380 mA. DNA was crosslinked to the membrane using a Stratagene Stratalinker UV 1800 Crosslinker. Detection was performed using the LightShift™ Chemiluminescent EMSA Kit, following the manufacturer’s protocol. Membranes were imaged on a Vilber Lourmat Fusion FX imaging system.

### Live microscopy

#### Brightfield imaging

Bright-field images of whole embryos were taken with a stereomicroscope (Olympus, SZX-12) equipped with a CCD camera. The embryos were transferred to a glass dish containing embryo medium and manually dechorionated using forceps prior to image acquisition. They were then positioned using forceps and a needle, taking care not to injure or pierce the embryos, and imaged in a glass dish.

#### Mounting embryos for spinning disk microscopy

When embryos reached the 32–64 cell stage, they were transferred to a glass dish containing embryo medium and manually dechorionated using forceps prior to mounting for imaging. Mounting medium was prepared by making a solution of 0.8% low-melting agarose in Danieau’s, containing 15% v/v OptiPrep (Sigma–Aldrich, D1156). The mounting medium was melted at 70°C and then cooled to 37°C in a glass vial until the embryos were ready to be mounted. The dechorionated embryos were transferred to the mounting medium at 37°C and mounted on an ibidi glass bottom μ-dish (Ibidi, 81158-400). The embryos were imaged using a Nikon CSU1 Yokogawa spinning disk microscope in a temperature-controlled chamber (Okolabs temperature controller and Nikon temperature-controlled chamber) using a Nikon SR HP Plan Apo 100×/1.35 Sil WD 0.3 objective. Serial optical sections were obtained for a z-stack of 30 μm, using intervals of 0.3 μm, with a time resolution of 3 min between sequential acquisitions.

### Image processing and analysis

#### Software

All microscopy images were viewed and analyzed using Fiji (Fiji is just ImageJ) [41]. Cluster segmentation and analysis were performed using the Fiji plugin “3D Objects Counter” [42]. Figures were made using the Fiji plugin “ScientiFig” [43].

#### Cluster segmentation with Fiji

Individual nuclei at the indicated time points were segmented in 3D with a 250 × 250 pixel bounding box using Fiji. The resulting files were named with a nomenclature that allowed the nuclei to be traced back to the original files they were segmented from. TF clusters were segmented using the 3D objects counter plugin in Fiji [42] and manually curated. Clusters were segmented at the midpoint between two mitoses, except for Sox19b and its deletions and NanogΔN-mNG, for which clusters were segmented right after mitosis because these only form transiently. For full-length Sox19b-mScarlet-I and Sox19bΔN-mScarlet-I, which form only two clusters, clusters were counted manually, using maximum intensity projections (MIPs) of whole nuclei.

#### Determining developmental stage in microscopy images

The embryonic developmental stages in microscopy images were determined on the basis of synchronicity of cell cycles, cell size, nuclear size, and inter-nuclei distance. The inter-nuclei distances provide a reliable method to determine cell sizes and can be found here (https://doi.org/10.5281/zenodo.8151889). The midpoint of each cell cycle was determined by measuring the time between two consecutive metaphases and taking the midpoint between these two timeframes.

#### Quantification of nuclear-to-cytoplasmic ratio

To calculate the nuclear-to-cytoplasmic ratio of fluorescence intensity, single z-slices from 3D confocal stacks were used. Regions of interest (ROIs) were drawn within the nucleoplasm of several individual nuclei, carefully avoiding any visible clusters, to sample fluorescence intensity representative of the diffuse nuclear signal. For each nucleus, five ROIs were sampled across different z-slices spanning the full depth of the nucleus. The average intensity of these five nuclear ROIs was used as the nuclear intensity for that cell. Corresponding cytoplasmic intensity was calculated by sampling five cytoplasmic ROIs from the same cell across the *z*-axis, again avoiding any bright puncta or outliers. The average of these five cytoplasmic measurements was used as the cytoplasmic intensity for that nucleus. The nuclear-to-cytoplasmic ratio was calculated as the average nuclear intensity over the average cytoplasmic intensity. This ratio was computed for each cell, and the resulting values were plotted for all constructs.

#### Quantification of Nanog enrichment in nuclear clusters

To determine the enrichment of Nanog within nuclear clusters, image stacks were analyzed in 3D using the 3D Objects Counter plugin in ImageJ. Nuclear clusters were identified and segmented in 3D, and the mean fluorescence intensity of mNeonGreen signal within all detected clusters in a given nucleus was calculated. Separately, the mean intensity of the surrounding nucleoplasm (excluding clusters) was measured by sampling cluster-free regions within the same nucleus. Enrichment was calculated as the ratio of fluorescence intensity inside clusters to that of the nucleoplasm as follows: cluster enrichment = mean intensity in clusters/mean intensity in nucleoplasm. This value was computed for each nucleus and plotted for all experimental conditions.

#### Comparing cluster number in diploid and tetraploid embryos

To determine if the fold difference in the number of clusters between diploid and tetraploid embryos deviates from 2, we log_2_ transformed the data, determined the mean values, and asked if the difference between them was significantly different than 1. Using the Mann–Whitney–Wilcoxon test, we found the *P*-values to be 6.3 × 10^−5^ for Nanog, 0.77 for Pou5f3, and 0.298 for Sox19b.

#### Sample size for imaging experiments

All experiments were performed keeping a minimum of three independent biological replicates. In these three biological replicates, multiple embryos—typically three or more per replicate—were included, consistent with standard practices in the field. The total number of embryos is represented by *N*. A minimum of 25 nuclei were selected for analysis (*n* > 25). The nuclei were evenly distributed across embryos from all replicates to ensure balanced representation in the analysis. The details for the number of embryos (*N*) and number of nuclei (*n*) for each experiment are listed in the table below.

**Figure 1.**
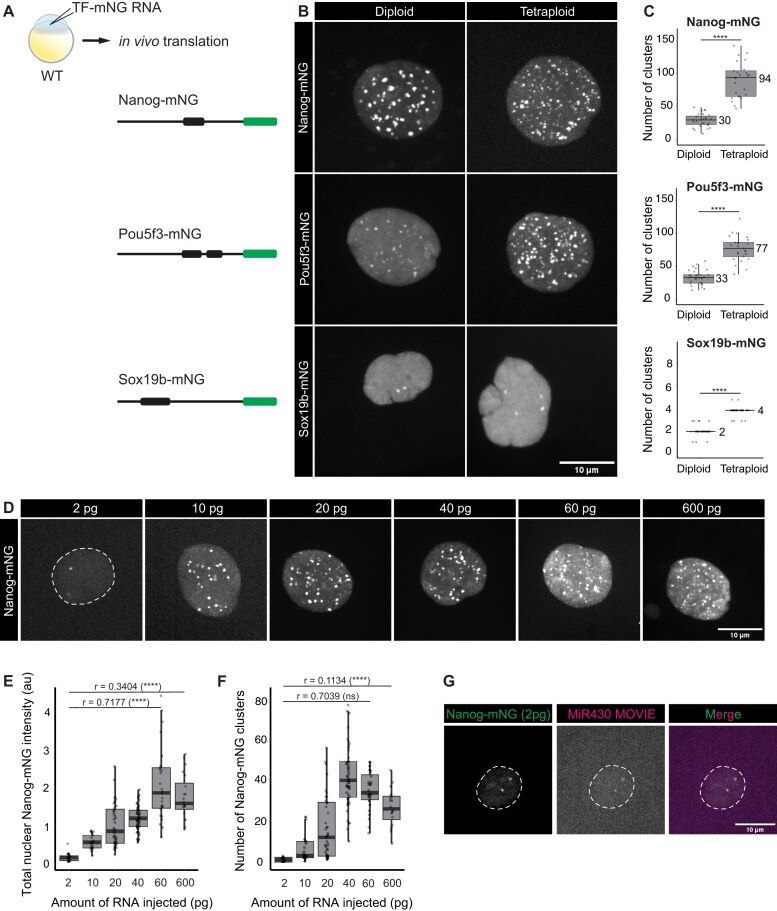
TFs form clusters in the nucleus, and these are DNA-seeded. (**A**) RNA coding for Nanog-mNG, Pou5f3-mNG, and Sox19b-mNG was injected in one-cell stage WT embryos. The schematics represent the translated protein products, where the TF is represented in black (boxes are DBD, lines are N- and C-terminal IDRs) and mNeonGreen is represented in green. Images were taken at 512-cell stage at the midpoint between two mitoses, except for Sox19b for which the image was taken right after mitosis because clusters only form transiently. (**B**) Visualization of Nanog-mNG, Sox19b-mNG, and Pou5f3-mNG in diploid and tetraploid embryos. (**C**) Quantification of the number of clusters per nucleus in diploid and tetraploid WT embryos. The median values of the distributions are indicated in the graphs. We used a Mann–Whitney test to compare the medians and calculate the *P*-values. Quantifications for additional stages are shown in Supplementary Fig. S1A. With *N* as the number of embryos and *n* as the total number of nuclei, *N* ≥ 9 and *n* ≥ 25 (see the “Materials and methods” section for more details on replicates). (**D**) Visualization of Nanog-mNG clusters after injecting WT embryos with a concentration series of Nanog-mNG RNA, as indicated. (**E**) Quantification of total nuclear intensity of mNeonGreen in individual nuclei as a proxy for total nuclear Nanog protein at different concentrations of injected RNA. au = arbitrary units. We calculated Pearson’s correlation coefficients (*r*) as indicated in the graph. A linear correlation is observed up to 60 pg (*r* = 0.7177), but this relationship breaks down when 600 pg of RNA is included (*r* = 0.3404). (**F**) Quantification of the number of clusters per nucleus at different concentrations of injected RNA. We calculated Pearson’s correlation coefficients (*r*) as indicated in the graph. A linear correlation is observed up to 60 pg (*r* = 0.7039), but this relationship breaks down when 600 pg of RNA is included (*r* = 0.1134). With N as the number of embryos and *n* as the total number of nuclei, *N* ≥ 8 and *n* ≥ 25. (**G**) Visualization of the overlap between Nanog-mNG and MiR430 RNA in WT embryos that were injected with 2 pg of Nanog-mNG RNA. In panels (B), (C), and (E), MIPs in Z of representative individual nuclei extracted from spinning disk confocal microscopy at the 512-cell stage are shown.

**Figure 2.**
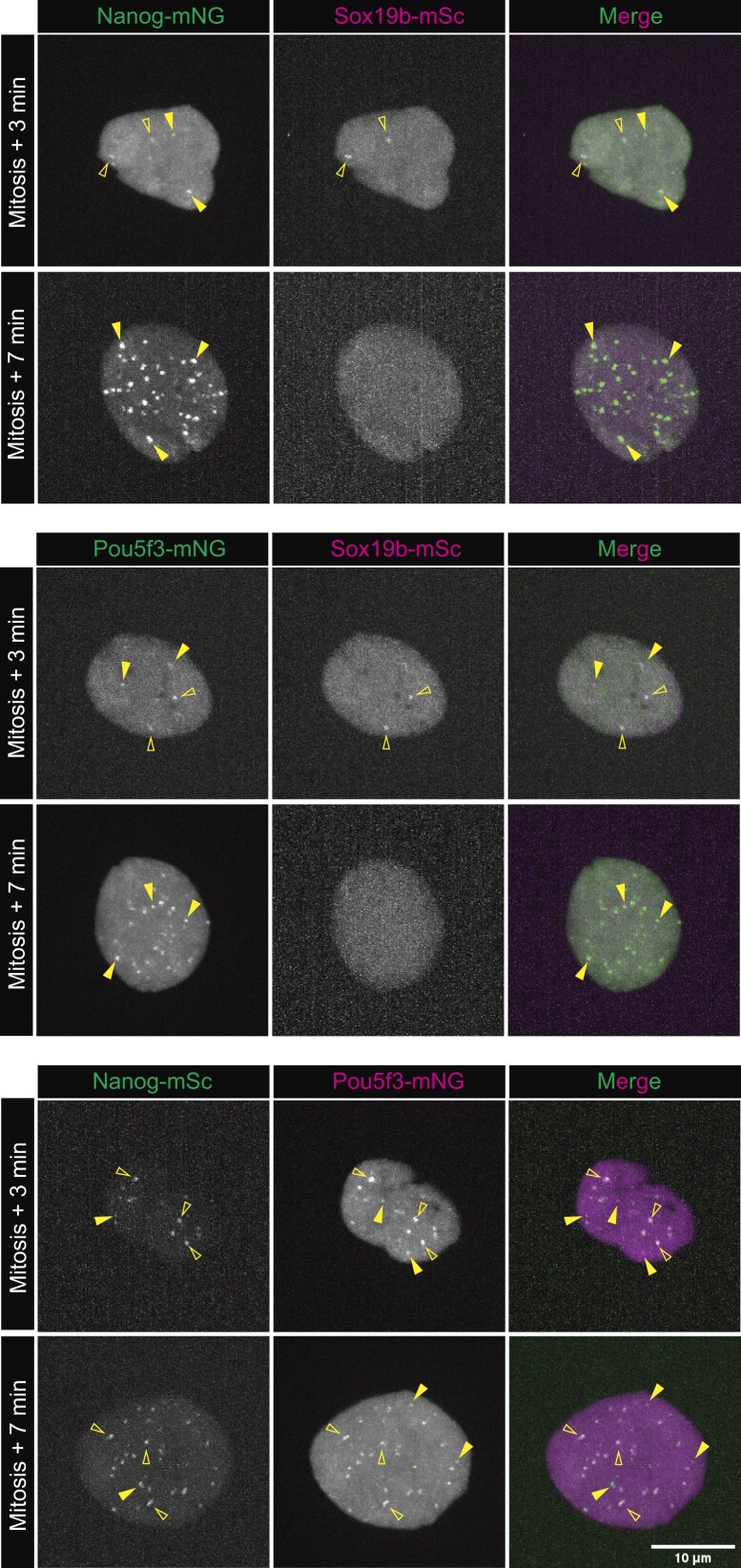
Nanog, Sox19b, and Pou5f3 cluster in specific combinations. Pairwise visualization of TFs as indicated. Shown are representative images of MIPs in z of individual nuclei extracted from spinning disk confocal microscopy at 512-cell stage. Images were taken at 3 min after mitosis (just as the nucleus is reforming) and 7 min after mitosis (midpoint between two mitoses). Since Sox19b clusters only exist transiently, they can only be visualized 3 min after mitosis. Nonoverlapping examples are indicated with filled arrowheads, and overlapping examples are indicated with empty arrowheads. With *N* as the number of embryos and *n* as the number of nuclei, *N* ≥ 8 and *n* ≥ 25.

**Figure 3.**
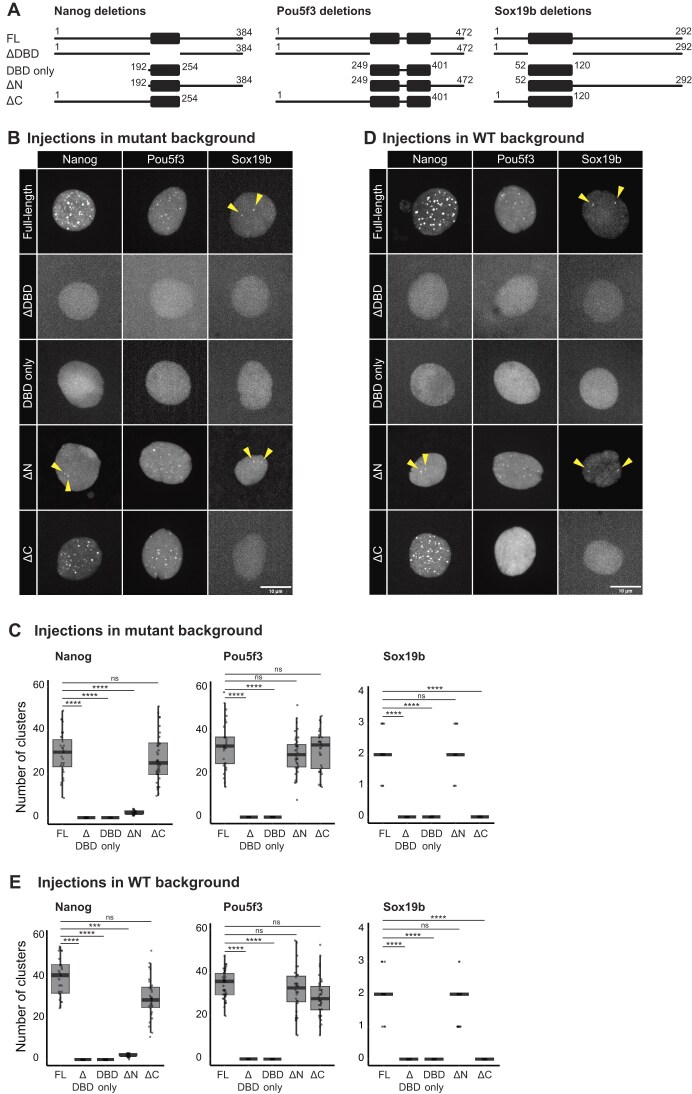
DBD is required to join an existing cluster. (**A**) Schematic representation of the deletion constructs that were injected into one-cell stage embryos. Numbers indicate the amino acids that were retained. DBD is shown as a box. (**B**) Images obtained after injection of the indicated constructs in the respective TF MZ mutants. Arrowheads indicate the two clusters formed by NanogΔN-mNG, full-length Sox19b, and Sox19b deletions. Shown are representative images of MIPs of individual nuclei at interphase extracted from spinning disk confocal microscopy at 512-cell stage. Images were taken at the midpoint between two mitoses, except for full-length Sox19b, Sox19b deletions, and NanogΔN-mNG, for which the image was taken right after mitosis because these clusters only form transiently. (**C**) Quantification of the number of clusters shown in panel (B). With *N* as the number of embryos and *n* as the number of nuclei, *N* ≥ 7 and *n* ≥ 28. (**D**) As in panel (D), but after injection of the indicated constructs injected in WT embryos. (**E**) Quantification of the number of clusters represented in panel (D). With *N* as the number of embryos and n as the number of total nuclei, *N* ≥ 7 and *n* ≥ 28. Statistical analysis in panels (C) and (E) was performed using Kruskal–Wallis test with Dunn’s multiple comparisons.

**Figure 4.**
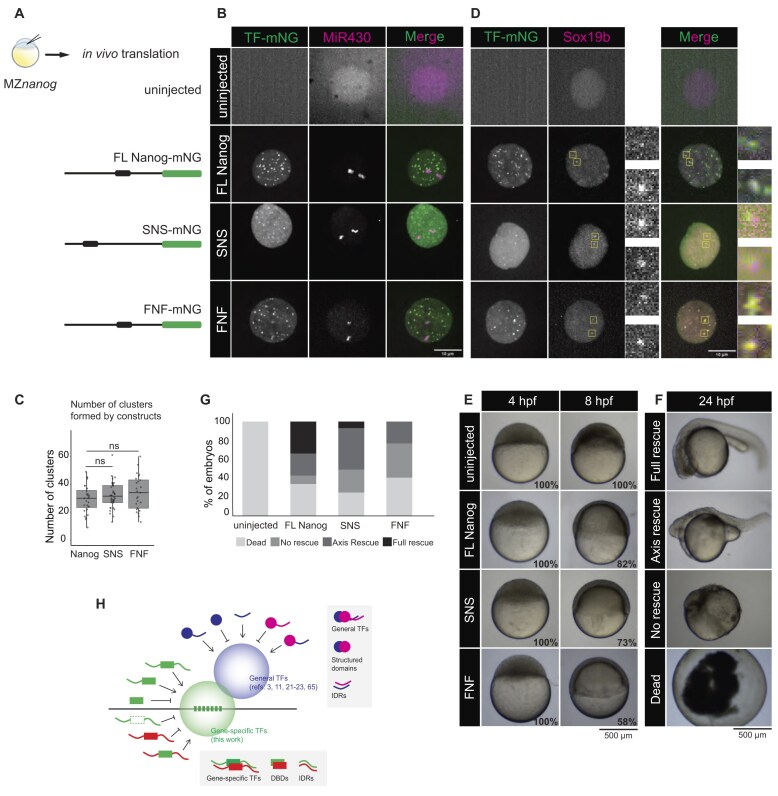
Specificity in TF clustering is mediated by the DBD. (**A**) RNA coding for indicated proteins was injected in one-cell stage MZ*nanog* embryos. The schematics represent the translated protein products, where the TF is represented in black (boxes are DBD, lines are N- and C-terminal IDRs) and mNeonGreen is represented in green. SNS and FNF refer to chimeric proteins as described in the text. (**B**) Visualization of the indicated TFs and MiR430 RNA in MZ*nanog* embryos. Shown are representative images of MIPs of individual nuclei at interphase extracted from spinning disk confocal microscopy at 512-cell stage. (**C**) Quantification of the number of Nanog-mNG, SNS-mNG, or FNF-mNG clusters per nucleus in MZ*nanog* embryos. With *N* as the number of embryos and *n* as the number of total nuclei, where *N* ≥ 9 and *n* ≥ 25. Statistical analysis was performed using Kruskal–Wallis test with Dunn’s multiple comparisons. (**D**) Visualization of the indicated TFs and Sox19b in MZ*nanog* embryos. The boxed regions highlight the two Sox19b clusters, with the inset providing a zoomed-in view. Shown are representative images of MIPs, as in panel (B). (**E**). Embryonic phenotypes of MZ*nanog* embryos injected with the indicated RNA. Shown are representative images of individual embryos captured at 4 and 8 hpf. The percentages indicate the fraction of embryos with the shown phenotype. (**F**) Classification of the embryonic phenotypes observed at 24 hpf, as described in [28]. (**G**) Classification of embryos at 24 hpf upon injection of chimeric constructs. Categories are described in panel (F). No uninjected embryos survived to reach 24 hpf; they all died during epiboly. With *N* as the number of embryos and *n* as the number of total embryos, *N* = 3 and *n* > 180 embryos. (**H**) Schematic model for the formation of a transcription body. Gene-specific TFs cluster on specific DNA sequences, targeted by their DBD and facilitated by their IDRs. This results in a local high concentration of gene-specific TFs that facilitates the recruitment of the general transcription machinery. The specificity in clustering of general TFs is mediated by IDRs, resulting in the clustering of proteins with similar IDR composition. This model allows for the sequence-specific clustering of gene-specific TFs, while facilitating the recruitment of a set of general TFs that are universally required for transcription.

## Information on the number of embryos (*N*) and number of nuclei (*n*) per figure

**Table:** 

Figure	Category	*N*	*n*
1B	Nanog diploid	10	28
	Nanog tetraploid	10	25
	Pou5f3 diploid	9	30
	Pou5f3 tetraploid	13	25
	Sox19b diploid	12	38
	Sox19b tetraploid	9	30
1D	2 pg	8	25
	10 pg	9	25
	20 pg	11	45
	40 pg	14	53
	60 pg	9	27
	600 pg	10	25
Supplementary Fig. S1	Nanog 256-cell	11	24
	Nanog 1k-cell	9	23
	Pou5f3 256-cell	9	24
	Pou5f3 512-cell	9	30
	Pou5f3 1k-cell	10	35
	Sox19b 256-cell	6	12
	Sox19b 512-cell	6	16
	Sox19b 1k-cell	9	28
2	Nanog(-mNG) and Sox19b(-mSc)	8	25
	Nanog(-mSc) and Pou5f3(-mNG)	11	25
	Pou5f3(-mNG) and Sox19b(-mSc)	10	25
3C	Nanog FL	8	27
	NanogΔHD	9	30
	Nanog HD only	8	30
	NanogΔN	8	30
	NanogΔC	10	31
	Pou5f3 FL	9	39
	Pou5f3ΔDBD	10	30
	Pou5f3 DBD only	8	30
	Pou5f3ΔN	11	32
	Pou5f3ΔC	7	39
	Sox19b FL	9	31
	Sox19bΔHMG	9	30
	Sox19b HMG only	10	30
	Sox19bΔC	9	30
3E	Nanog FL	10	28
	NanogΔHD	7	30
	Nanog HD only	9	30
	NanogΔN	8	26
	NanogΔC	8	40
	Pou5f3 FL	9	30
	Pou5f3ΔDBD	8	30
	Pou5f3 DBD only	9	30
	Pou5f3ΔN	7	39
	Pou5f3ΔC	9	32
	Sox19b FL	11	38
	Sox19bΔHMG	7	30
	Sox19b HMG only	9	30
	Sox19bΔN	10	30
	Sox19bΔC	8	30
4C	Nanog FL	9	28
	SNS chimera	11	40
	FNF chimera	9	25
4G	Nanog FL	3	180
	SNS chimera	3	275
	FNF chimera	3	389
	*nanog ^−/−^* uninjected	3	330

### Data plotting and statistical analysis

Image analysis files in .csv format were exported to RStudio software for data plotting and visualization. Graphs were generated using the library ggplot2 [44]. All statistical analysis was performed using GraphPad Prism. Nonparametric statistical tests, Mann–Whitney test (for two groups) or Kruskal–Wallis test with Dunn’s comparisons (for multiple comparisons) were performed to calculate the *P* values for significance. Statistical significance was reported using a four-star system, with *P* < .05 indicated by (*), *P* < .01 by (**), *P* < .001 by (***), *P* < .0001 by (****), and nonsignificant results (*P* ≥ 0.05) denoted as ns.

## Results

To investigate the clustering of TFs, we focused on Nanog, Pou5f3, and Sox19b, all of which play important roles in the activation of transcription in the zebrafish embryo [27–30]. We injected RNA encoding each of these TFs fused to mNeonGreen (mNG in figures) into one-cell stage WT embryos (Fig. 1A). For Nanog and Pou5f3, we used the concentration that was shown to rescue the MZ mutant phenotype [28, 34]. For Sox19b, the MZ mutant is viable, so here we used the same number of moles of RNA as used for Nanog, for consistency. We visualized the distribution of TFs in the nucleus by spinning disk microscopy (Fig. 1B, diploid). All three TFs form clusters in the nucleus, as previously reported [17, 26, 45, 46]. Quantification of the number of clusters at the 512-cell stage, when clusters are abundant and clearly visible, showed that Nanog, Pou5f3, and Sox19b form distinct numbers of clusters (Fig. 1C, diploid). We detect a median of 30 Nanog clusters per nucleus. The number of Pou5f3 clusters is similar, with a median of 33, and Sox19b generally forms only two clusters, and only transiently. These numbers lie in a similar range at the 256- and 1k-cell stages (Supplementary Fig. S1A).

The concentration of a specific TF in the nucleus has been shown to influence the number of clusters that form [6, 46, 47]. To directly test the relationship between TF concentration and cluster number, we injected between 2 and 600 pg of RNA encoding Nanog-mNG into MZ*nanog* embryos (Fig. 1D). To determine whether the increase in injected RNA resulted in an increase in protein, we then measured the total intensity of nuclear mNeonGreen in all conditions, using this as a proxy for the total amount of Nanog protein in the nucleus. While an increase in RNA initially results in an increase in protein, no further increase in the amount of protein is observed between 60 and 600 pg of injected RNA (Fig. 1D and E). We speculate that this might be due to saturation of the translational machinery. We then quantified the number of clusters that form with an increasing amount of RNA, as well as the fraction of nuclear Nanog protein that is in clusters. As we increased from 2 to 40 pg of injected RNA, the number of clusters in the nucleus increased. Above this concentration, the number of clusters stabilized and even decreased (Fig. 1D and F). The fraction of fluorescence in clusters increases slightly with increasing concentration (Supplementary Fig. S1B). We conclude that an increase in the amount of protein in the nucleus results in an increase in the number of clusters that form.

The clustering of TFs may reflect the formation of protein aggregates in the nucleoplasm [47] or the formation of clusters that are DNA-seeded [10, 48–52]. To distinguish between these two possibilities, we took advantage of the ease of making tetraploid zebrafish embryos [35] (see the “Materials and methods” section). If clusters are TF aggregates, a doubling of DNA would be predicted to have a negligible effect on the number of clusters at the same TF concentration. If clusters are DNA-seeded, however, it would be predicted to roughly double the number of TF clusters. We injected tetraploid embryos with the same constructs at the same concentration as the diploid embryos and observed that the number of clusters increased significantly in tetraploid embryos (Fig. 1B). Comparing the number of clusters between diploid and tetraploid showed a doubling in the number of clusters for Pou5f3 and Sox19b, while the number of Nanog clusters showed a larger increase (Fig. 1C; see the “Materials and methods” section for approach and statistics). These results show that the number of clusters is correlated with the DNA content. We conclude that the Nanog, Pou5f3, and Sox19b clusters are DNA-seeded.

Remarkably, we see two Nanog clusters even when injecting only 2 pg of RNA (Fig. 1D). We and others have previously shown that two of the Nanog clusters in the nucleus form at the *mir430* locus [17, 26, 45]. This locus has many binding sites for Nanog [27, 53] and can thus be considered a high-affinity region. In line with this, the Nanog clusters that form on the *mir430* locus are the first to appear when cells exit mitosis [17]. We therefore hypothesized that the two Nanog clusters that form when injecting 2 pg of Nanog RNA form at the *mir430* locus. Visualization of MiR430 transcripts using Morpholino VIsualization of Expression [36] (MoVIE) confirmed this (Fig. 1G). We conclude that the concentration of Nanog affects the number of clusters that form and that Nanog clusters first at high-affinity sites.

The model for gene-specific TF clustering that has been put forward postulates that TFs bind sequence-specifically to DNA, after which additional factors can join by means of their IDR. In the very simple scenario in which there is no specificity in the interactions between IDRs, this would predict that once a TF is bound to DNA, any other protein with an IDR could associate with this TF and contribute to cluster formation. If this were the case, TF clusters would be predicted to contain all TFs with IDRs, regardless of their DNA-binding specificity. To directly assess the specificity in TF clustering, we asked whether the Nanog, Sox19b, and Pou5f3 clusters always overlap (nonspecific clustering) or form clusters in different combinations (specific clustering). To do so, we generated the constructs shown in Fig. 1A but now with the fluorescent protein mScarlet-I (mSc in Figures), which does not spectrally overlap with mNeonGreen. Co-injecting the mNeonGreen and mScarlet-I constructs, we could visualize two TFs at the same time and assess their co-localization (Fig. 2). Sox19b-mSc forms two clusters (as also seen with mNeonGreen, Fig. 1B), and these colocalize with Nanog as well as with Pou5f3 (empty arrowheads). These are the clusters that form at the *mir430* locus [26]. Both Nanog and Pou5f3 form many more clusters (especially at mitosis + 7min, when the transient Sox19b clusters are no longer visible), and these do not colocalize with Sox19b (Fig. 2, filled arrowheads). Similarly, when assessing the overlap between Nanog and Pou5f3 clusters, we observe clusters that are Nanog-only and Pou5f3-only (Fig. 2, filled arrowheads). We conclude that there is a high level of specificity in the co-clustering of Nanog, Sox19b, and Pou5f3.

We then set out to investigate what mediates the specificity in clustering of Nanog, Sox19b, and Pou5f3. The three proteins show the typical structure of a TF with a highly structured DBD and disordered regions of variable length that flank this domain (Supplementary Fig. S2A; see the “Materials and methods” section). The Nanog DBD [homeodomain (HD)] is flanked by two IDRs of similar size. The Sox19b DBD (HMG domain) is also flanked by two IDRs: one shorter N-terminal IDR and a longer C-terminal IDR. Similarly, the Pou5f3 DBD (there are two: Pou and homeodomain) is flanked by a long N-terminal IDR and a shorter C-terminal IDR. To investigate the role of DBD and IDRs in cluster formation, we created constructs in which each of them was deleted or mutated (Fig. 3A and Supplementary Fig. S2B) and verified the expression level and nuclear localization of the encoded proteins upon injection of RNA into zebrafish embryos (Supplementary Fig. S3).

We distinguish the seeding of a cluster (which refers to the binding of a TF to the DNA) from the growth of a cluster (which refers to the joining of additional factors to an already existing cluster). First, to investigate which domains are important to form a cluster (both seeding and growth), we injected the constructs we generated into embryos that are MZ mutant for the injected TF so that no endogenous full-length protein is present to facilitate clustering [28, 34]. We first injected Nanog without its DNA binding domain (ΔDBD) in MZ*nanog* embryos. This did not result in the formation of any clusters (Fig. 3B and C). This shows that, as expected, the DBD is required for the formation of Nanog clusters. This is specifically due to the loss of its DNA-binding capacity, because a point mutant that abrogates DNA-binding gives the same result (Supplementary Fig. S2B and C). The DBD, while required and by itself sufficient to bind to DNA [as evidenced by its mitotic retention (Supplementary Fig. S4) and in gel-shift assays (Supplementary Fig. S5)], is not sufficient for cluster formation, because we see no clusters when we inject the Nanog DBD-only construct (Fig. 3B and C). Thus, both the DBD and the IDRs are required for cluster formation. This is in line with the existing model. We then tested the role of the N- and C-terminal IDR of Nanog separately. Interestingly, the deletion of the N-terminal IDR resulted in a drastic reduction in the number of clusters, as only two clusters per nucleus were observed on average (Fig. 3B and C). These form at the *mir430* locus (Supplementary Fig. S2D). Because the N-terminal IDR has been shown to harbor a dimerization domain [54], however, it is unclear whether the importance of this IDR is caused by the loss of a single IDR or the loss of the dimerization domain. We therefore turned to the results obtained after deletion of the C-terminal IDR. Here, we find that clusters still form, and their numbers are comparable with those for full-length Nanog (Fig. 3B, C). We conclude that for Nanog, an IDR or dimerization domain in addition to the DBD is sufficient to form clusters. We then injected different variants of Pou5f3 in MZ*pou5f3* mutant embryos [34]. Again, we find that DBD as well as IDRs are important for clustering (Fig. 3B, C). Here, both IDRs are individually dispensable for clustering, suggesting that one IDR in addition to the DBD, is sufficient to generate clusters. Finally, we repeated these experiments for Sox19b, using MZ*sox19b* embryos [34]. These experiments confirmed that both the DBD and IDRs are important for clustering (Fig. 3B, C). Interestingly, in this case, the deletion of the short IDR (N-terminal) had no effect on clustering, while the removal of the longer IDR (C-terminal) abrogated clustering. This might suggest that IDR length matters for the ability of TFs to cluster. We note here, though, that even full-length Sox19b forms only two clusters, so the dynamic range of our analysis is not as good as for Nanog and Pou5f3. Taken together, we conclude that for Nanog, Sox19b, and Pou5f3, both DBD and at least one IDR are required to form a cluster. This is in line with a model in which the DBD is required to seed a cluster, and IDRs are required to grow it.

Next, we set out to investigate whether IDRs are sufficient to join a cluster, as would be predicted by the model. If true, it would be expected that any TF that retains either one or both IDRs would integrate into an existing cluster. To test this, we repeated the experiment described above, but now instead of injecting the constructs into mutant embryos, we used WT embryos, in which clusters of endogenous full-length protein are present [45, 46]. As expected, full-length Nanog is seen in clusters (Fig. 3D and E). The number of clusters lies in the same range as was seen for the mutant embryos (Fig. 3C and E), suggesting that the injected Nanog colocalizes with existing clusters. The homeodomain only (lacking both IDRs), in contrast, was unable to join clusters. The presence of an IDR along with the homeodomain restored the ability to join a cluster, with—as above—a stronger effect of the N- than the C-terminal IDR (Fig. 3D and E). Thus, to join a cluster, IDRs are indispensable, as would be predicted by the model. Contrary to expectations, however, we observed that neither NanogΔHD nor Nanog HD R247A were able to join existing clusters (Fig. 3D and E and Supplementary Fig. S2E). This shows that the Nanog DBD is required for Nanog to join an existing cluster, and IDR–IDR interactions alone are not sufficient. Experiments with Sox19b and Pou5f3 confirmed this (Fig. 3D and E). We conclude that for Nanog, Sox19b, and Pou5f3, the DBD of TFs is required even to join an existing cluster, which is in contrast with the current model for TF clustering.

The observation that the DBD of a TF is required to join an existing TF cluster suggests that specificity in cluster formation is encoded by the DBD and not the IDRs. To test this directly, we generated chimeric TFs in which the Nanog DBD is flanked by the IDRs of Sox19b (referred to as SNS) or the RNA-binding protein Fus (referred to as FNF) (Fig. 4A). We chose the Sox19b IDRs because we have shown that it co-clusters with Nanog (Fig. 2), indicating that the IDRs of these two TFs are compatible. We selected the Fus IDRs because they have been shown to facilitate clustering when fused to proteins [15], yet full-length zebrafish Fus does not form any clusters in the nucleus at 512-cell stage (Supplementary Fig. S6). These chimeric TFs were fused to mNeonGreen as before (Fig. 4A). We injected the chimeras and compared their clustering in MZ*nanog* embryos with that of full-length Nanog (Fig. 4B, C). We observed that the clustering of the SNS and FNF chimeras shows a stark difference from the clustering pattern of full-length Sox19b and full-length Fus (Fig. 1B and Supplementary Fig. S6). Instead, the chimeric proteins cluster like full-length Nanog (Fig. 4B), as confirmed by the quantification of the number of clusters (Fig. 4C). This suggests that the DBD determines where a chimeric protein clusters. To further test this, we generated a chimera that contained a Sox19bDBD flanked by Nanog IDRs (NSN). When injected into MZ*sox19b* embryos, we observed that this chimera formed only two clusters, which is the same as we observed for full-length Sox19b (Supplementary Fig. S7). Together this confirms that for Nanog and Sox19b, specificity in clustering is mediated by their DNA-binding domain, and it shows that IDRs can interact rather promiscuously when facilitating clustering.

In the chimera experiments, two of the SNS and FNF clusters form at the *mir430* locus, as visualized by labeling MiR430 transcripts (Fig. 4B). We and others have previously shown that Nanog is important to activate transcription from the *mir430* locus [26, 27]. The fact that we can see MiR430 transcripts when we inject chimeric proteins in a MZ*nanog* background shows that these chimeric proteins are not only able to cluster but also to activate transcription of the *mir430* locus, which makes them functionally similar to full-length Nanog. We have previously shown that at this locus, Nanog recruits Sox19b to activate transcription [26]. This would suggest that the chimeric proteins SNS and FNF are also able to recruit Sox19b. This is indeed the case because the SNS and FNF clusters both colocalize with Sox19b clusters (Fig. 4D, yellow boxes). Finally, we tested whether the chimeric constructs can also rescue the developmental arrest early during gastrulation seen in MZ*nanog* mutants. Interestingly, both the SNS and FNF chimeric constructs rescue the gastrulation phenotype, suggesting that they can functionally replace full-length Nanog not only at the *mir430* locus but also at other Nanog target genes (Fig. 4E). We have previously found that neither the Nanog-DBD-only nor the Nanog ΔDBD can rescue MZ*nanog* phenotype [26], so while the specific sequence of the IDRs does not seem to matter, the presence of the Nanog DBD and at least one IDR is required for the functional rescue at this developmental stage. The chimeras, however, fail to completely rescue the mutant phenotype at later stages of development (Fig. 4F and G). This may suggest that later in development the sequence of the IDR does matter, but we cannot exclude other possibilities such as, for example, the faster degradation of SNS and FNF than FL Nanog. We conclude that IDRs are important for clustering during early embryogenesis but are rather promiscuous in their interactions.

## Discussion

In this study, we show that the gene-specific TFs Nanog, Pou5f3, and Sox19b form clusters in the nucleus, that these clusters are DNA-seeded, and that their number scales with the amount of TF. As previously proposed, the DBD as well as at least one IDR are necessary to form a cluster. Surprisingly, however, the DBD is also required to join an existing cluster. Using chimeras, we confirm that the DBD determines the specificity of cluster formation and show that IDRs contribute to clustering through multivalent interactions, at least to a certain extent independent of their amino acid sequence.

### Binding site affinity and IDR–IDR interactions together promote the crowding needed for the clustering of gene-specific TFs

Our data show that both the DBD and the IDR of Nanog, Sox19b, and Pou5f3 are required for the formation of clusters on DNA (Fig. 3B and C). The importance of the DBD in clustering is undisputed when focusing on DNA-bound clusters [10, 48–51], although it might be dispensable when protein–protein interactions are abundant, as is—for example—the case for Zelda when it is recruited to GAF-rich regions in the *Drosophila* embryo [55]. The role of IDRs in the formation of TF clusters is less well understood. While IDRs certainly facilitate the clustering of TFs both *in vitro* [3, 11, 56] and *in vivo* [4, 15], they are not always required for cluster formation [48, 49, 52, 57, 58]. When IDRs are dispensable, the clusters are often seeded by a high density of closely spaced binding sites [48, 52, 58], which can probably bring together many proteins without the need of IDR-mediated interactions. Indeed, the number of TF binding sites has been shown to facilitate TF cluster formation [20, 59, 60]. Related to this, our work shows that the number of Nanog clusters that form depends on the concentration of a TF (Fig. 1E and F). This seems intuitive, and indeed, previous work in *Saccharomycescerevisiae* had found a similar relationship between protein concentration and the clustering of the yeast TF Gal4 [47]. In this study, however, not all the Gal4 clusters were associated with DNA, which made it difficult to uncouple the behavior of DNA-seeded clusters from non-DNA-bound clusters, especially because *in vitro* work has shown that the formation of non-DNA-bound clusters is also concentration dependent [20, 57]. In our study, Nanog clusters are DNA-bound (Figs. 1B and C and 3B and C, and Supplementary Fig. S2C). These DNA-bound TF clusters form preferentially at high-affinity binding sites (Fig. 1G), and, with increasing TF concentration, also form at lower-affinity sites. Taken together, this shows that the crowding that is needed for TF clustering can be promoted by high numbers of binding sites as well as protein–protein interactions mediated by IDRs.

### Specificity in the formation of gene-specific TF clusters is encoded in the genome

Our data show that once a Nanog, Sox19b, or Pou5f3 cluster has been seeded, additional molecules of the same TF can join the cluster only when a functional DBD is present (Fig. 3D and E, Supplementary Fig. S2E). This is in contrast with the current model that proposes that gene-specific TFs first bind to DNA via their DBD, followed by IDR-mediated recruitment of additional factors to create a cluster (see the “Introduction” section), as this model would predict that once a cluster has been seeded, additional molecules can join the cluster through IDR–IDR interactions. Our data show that this is not the case for Nanog, Sox19b, and Pou5f3 (Fig. 3 and Supplementary Fig. S2E). Thus, even though not all TFs in a cluster are bound to DNA at any given time [18, 26, 60], they all need to possess a functional DBD to join the cluster (Fig. 4H). This is confirmed by our experiments with chimeric proteins, which showed that the DBD, and not the IDR, determines where a chimeric protein will cluster (Fig. 4 and Supplementary Fig. S7). This implies that when co-clustering of different TFs is observed [6, 57, 61] (Fig. 2), this is driven by the presence of binding sites for the factors that are seen to co-cluster and not simply by IDR–IDR interactions. Indeed, at the *mir430* locus, where we see co-clustering of Nanog, Sox19b, and Pou5f3, binding sites for these factors have been identified [27, 36]. Extrapolating this to other clusters where multiple TFs come together, we assume that these represent other genomic regions where binding sites for these factors co-occur. We conclude that the clustering of gene-specific TFs is dictated by the genome, which ensures specificity in TF cluster formation (Fig. 4H).

### IDRs are promiscuous when facilitating the clustering of gene-specific TFs

Our chimera experiments show that IDRs of gene-specific TFs facilitate clustering, but that their specific protein sequence is not important for clustering (Fig. 4 and Supplementary Fig. S7). We note here that the Nanog and Sox19b IDRs that we tested in our chimeras are in principle compatible, as they can co-cluster endogenously. Fus, however, does not form clusters in nuclei of zebrafish embryos at the stage we analyzed (Supplementary Fig. S6). Therefore, especially the ability of Fus IDRs to functionally replace Nanog IDRs suggests that IDRs are rather promiscuous in cluster formation and transcription activation in the early embryo. It has been shown that IDRs can mediate specificity in the binding of gene-specific TF to DNA [24, 62, 63], so we cannot exclude the possibility that our observation is limited to the TFs we tested or early embryonic stages. The latter may be supported by our observation that the chimeric TFs can recapitulate the function of the WT TFs in the early embryo but fail to do so at later stages of development (Fig. 4E–G). It will be interesting to investigate in more detail how specific sequence features within the IDRs of gene-specific TFs influence their function, as has been done in yeast [64].

### Specificity in clustering in gene-specific TFs versus general TFs

Our data show that for the gene-specific TFs Nanog, Sox19b, or Pou5f3, specificity in clustering is dictated by their DNA-binding domain (Fig. 4H). This is in contrast with data on the clustering of general TFs in which specificity is mediated by IDRs [21, 22, 56, 65]. In these IDRs, specificity is encoded by distinct sequence features, and IDRs with similar features show similar partitioning [21, 22] (Fig. 4H). We propose a model in which both mechanisms of clustering coexist and represent different steps in the assembly of a transcription body. In this model, gene-specific TFs cluster on specific DNA sequences, targeted by their DBD and facilitated by their IDRs. This results in a local high concentration of gene-specific TFs that facilitates the recruitment of the general transcription machinery. The specificity in clustering of general TFs is then mediated by IDRs, resulting in the clustering of proteins with similar IDR composition. This would ensure the sequence-specific clustering of gene-specific TFs while facilitating the recruitment of a set of general TFs that are universally required to initiate transcription.

## Supplementary Material

gkaf625_Supplemental_File

## Data Availability

Raw imaging data are available upon request. All other data are available in the main text or the supplementary data.
