# Supplementary material for: Specificity in clustering of gene-specific transcription factors is encoded in the genome
Source: Nucleic Acids Res. 2025 Jul 12;53(13):gkaf625. doi: 10.1093/nar/gkaf625 (PMC12255298; doi:10.1093/nar/gkaf625)
Supplement: gkaf625_Supplemental_File [file gkaf625_supplemental_file.pdf]

Supplementary Figures

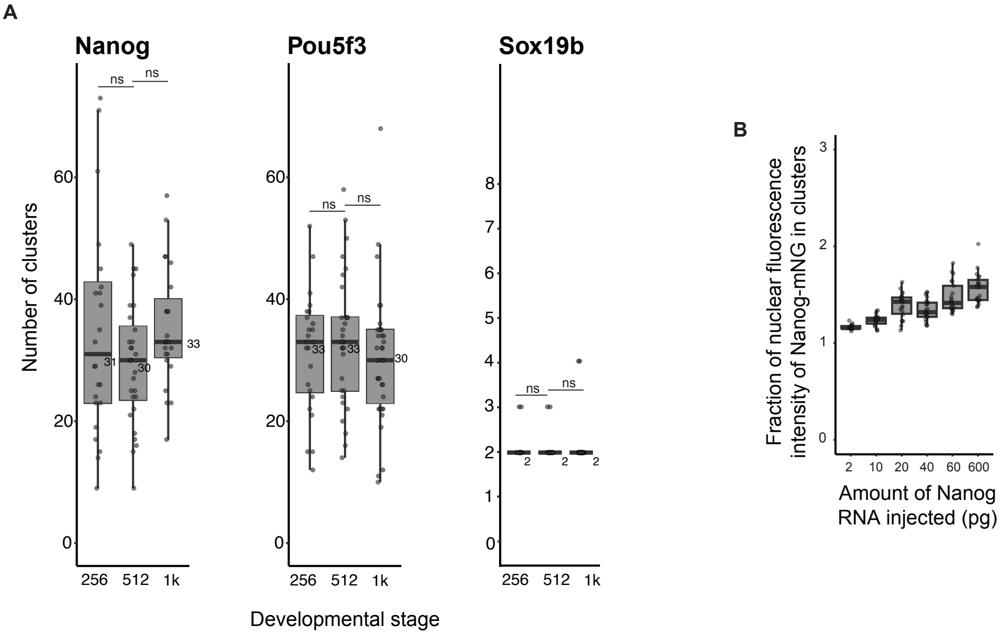

Figure S1. Quantification of TF clusters in zebrafish embryos at 256, 512 and 1k-cell stage. A. Quantification of the number of clusters for Nanog-mNG, Sox19b-mNG and Pou5f3-mNG at the 256-cell, 512-cell, and 1k-cell stage in WT TLAB embryos. The median values of the distributions are indicated in the graphs. With N as the number of embryos and n as the number of total nuclei, N ≥6 and n ≥12. Quantifications were performed at the midpoint between two mitoses, except for Sox19b for which the quantification was performed right after mitosis because clusters only form transiently. Statistical analysis was performed using Kruskal-Wallis test with Dunn's multiple comparisons. **B.** Quantification of the fraction of nuclear fluorescence intensity (Nanog-mNeonGreen) in clusters at the 512-cell stage for increasing amounts of injected RNA. With N as the number of embryos and n as the number of total nuclei, N ≥ 8 and n ≥ 20.

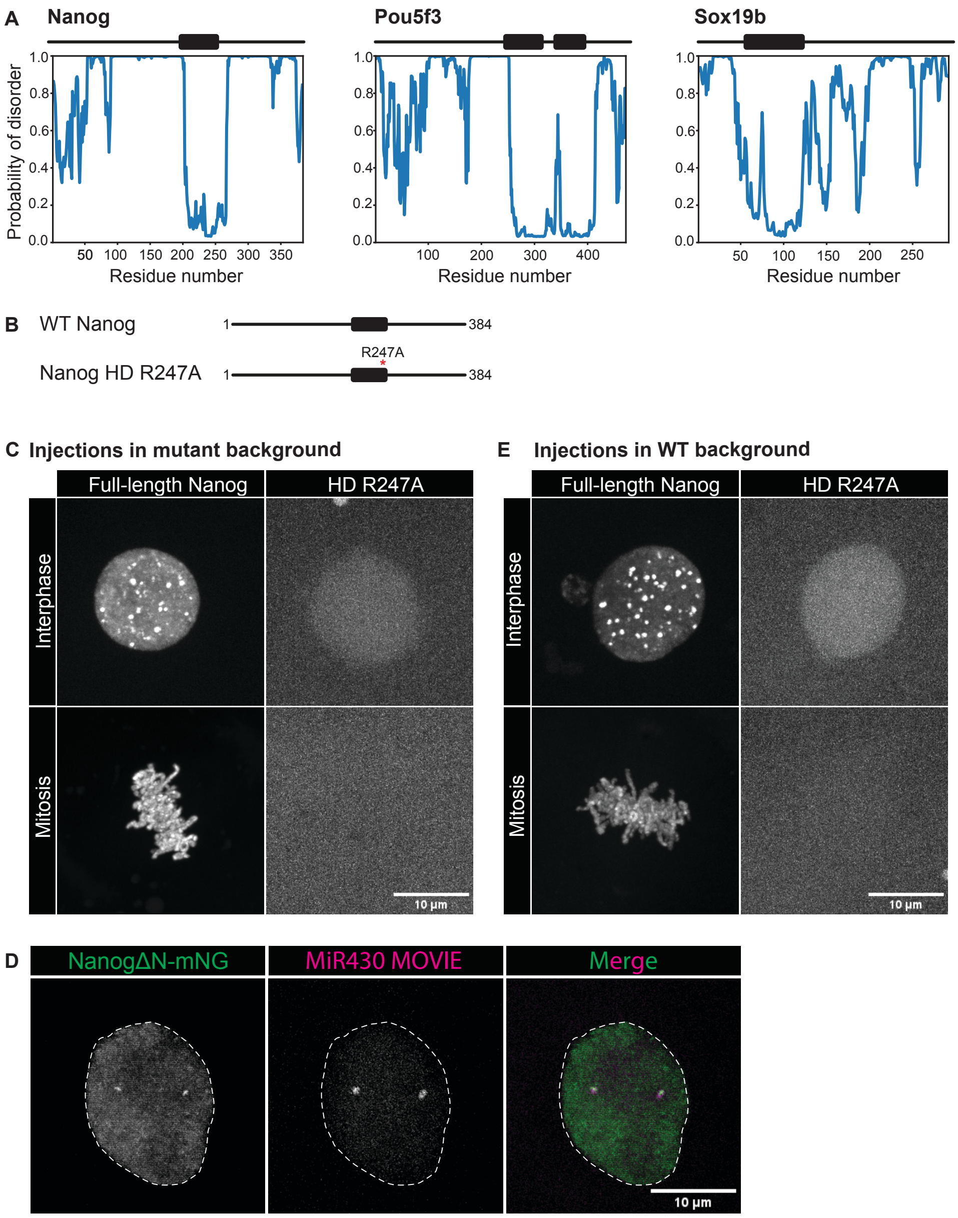

Figure S2. Additional information related to Figure 3. A. Disorder scan of Nanog, Pou5f3 and Sox19b generated using the ODINPred disorder prediction tool (1). See Methods for how ordered and disordered regions were defined. **B.** Schematic representation of full-length Nanog and the Nanog point mutant in which DNA binding is abrogated (Nanog HD R247A), for which RNA was injected in C and E of this Figure. **C.** Images of MZ*nanog* embryos injected with WT Nanog and Nanog HD R247A. Shown are representative images of individual nuclei extracted from spinning disk confocal microscopy at 512-cell stage during interphase and mitosis. **D.** Colocalisation of NanogΔN-mNG and MiR430 transcripts in WT embryos. The images are taken right after mitosis because NanogΔN-mNG clusters only from transiently. **E.** Images of WT embryos injected with WT Nanog and Nanog HD R247A. With N as the number of embryos, and n as the number of nuclei. N ≥6 and n ≥20. In C-E, representative images of MIPs in Z of individual nuclei extracted from spinning disk confocal microscopy at 512-cell stage are shown.

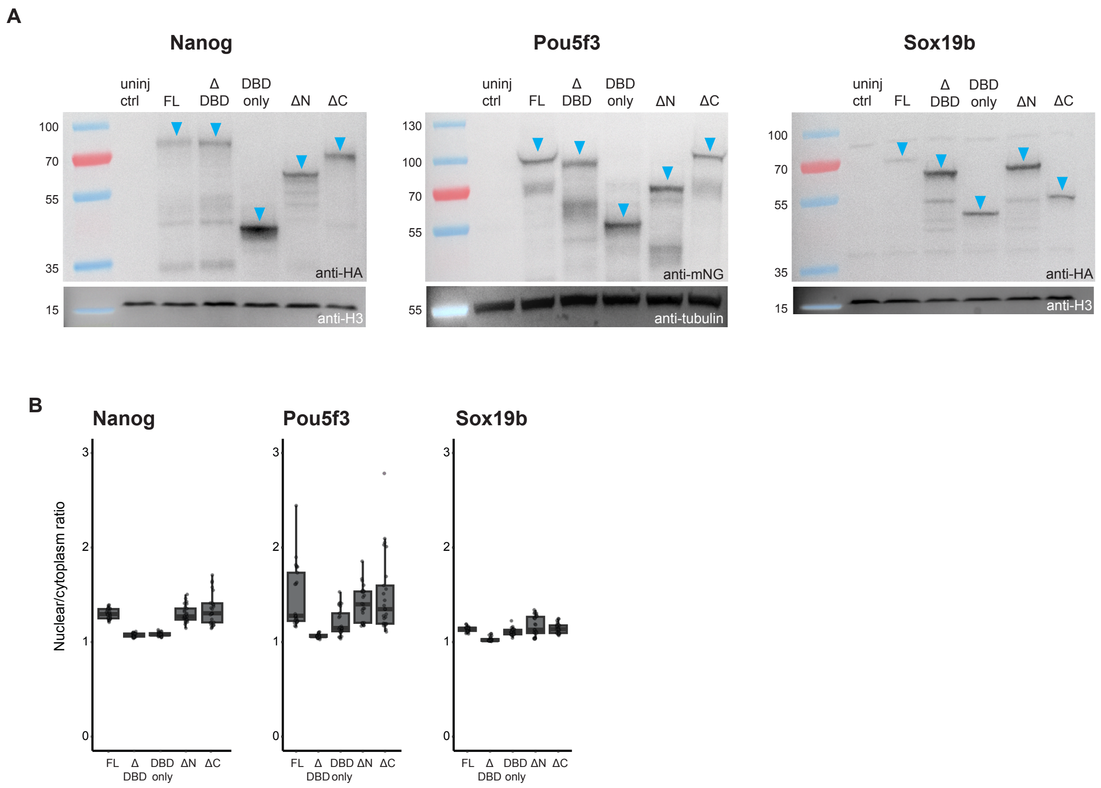

Figure S3. Expression levels and N/C ratio for the different versions of Nanog, Pou5f3 and Sox19b. A. Western Blots showing the expression level of the different versions of Nanog, Pou5f3 and Sox19b for which RNA was injected in WT embryos. Analyis was done at sphere stage. The first lane in each blot contains the molecular weight ladder, with band sizes (in kDa) indicated on the left. This is followed by the uninjected control embryos, and the indicated constructs. Blue arrowheads point to the protein of interest. Loading controls are shown in the lower panels: Shown is a representative example of n=2 biological replicates. **B.** Quantification of the nuclear to cytoplasm ratio for the same constructs as in panel A. With N as the number of embryos and n as the number of total nuclei, N ≥8 and n ≥25.

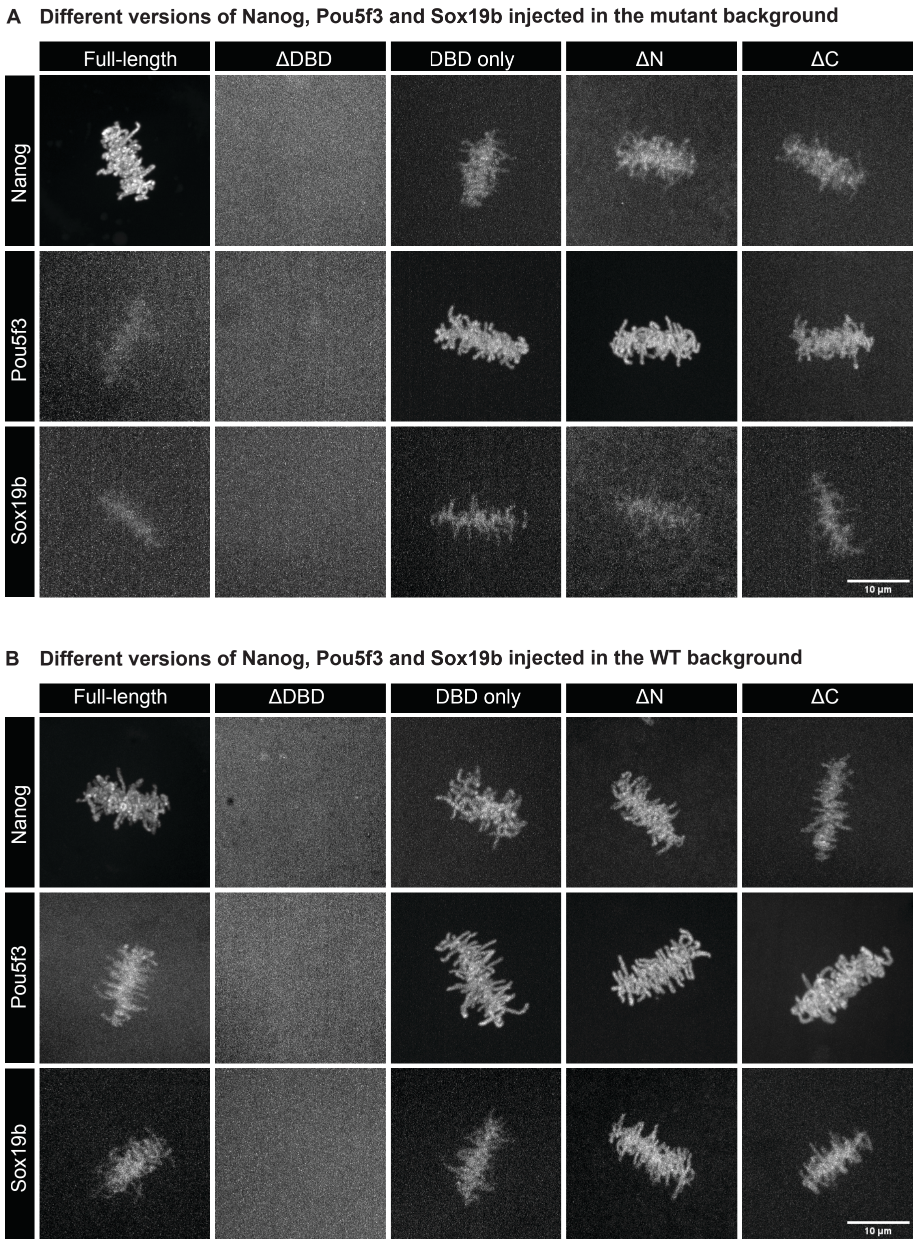

Figure S4. The binding of full-length and mutant Nanog, Pou5f3 and Sox19b proteins to DNA during mitosis. A. Images during mitosis of Nanog, Pou5f3 and Sox19B obtained after injection of the indicated constructs in the respective TF mutants. Related to Figure 3B, C. **B.** Same as in A but with constructs injected in a WT background, where endogenous protein of the injected factor is present. Related to Figure 3D, E. Shown are representative images of MIPs extracted from spinning disk confocal microscopy.

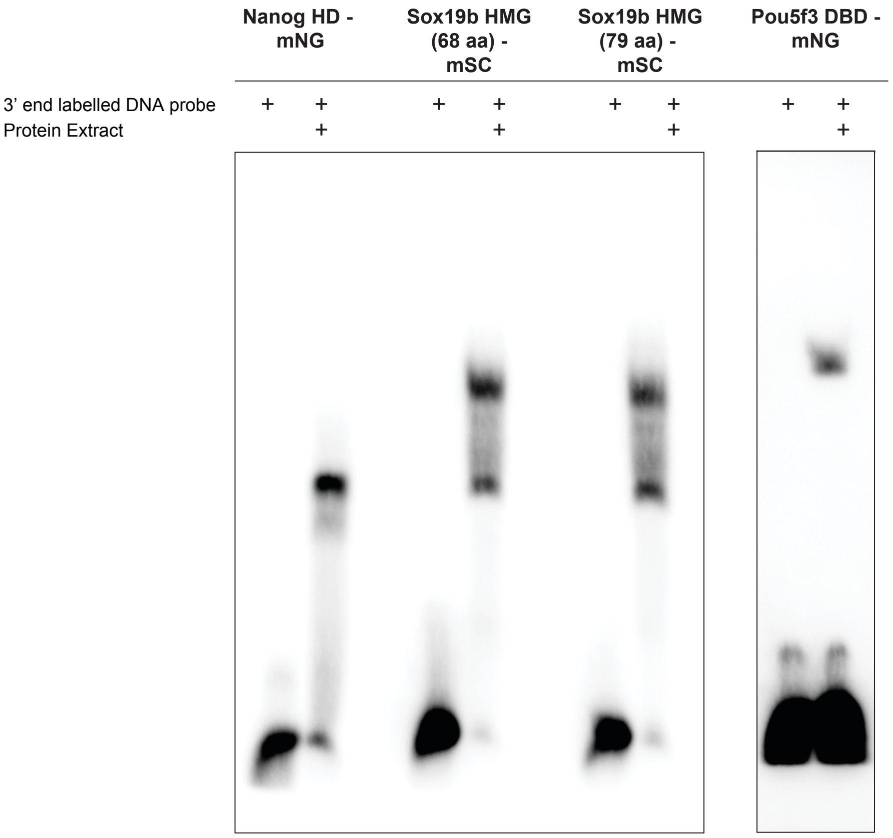

Figure S5. DBD only constructs fused to fluorescent protein can bind to DNA specifically. Electrophoretic Mobility Shift Assays (EMSAs) were performed using purified DNA-binding domains fused to fluorescent proteins (5 µg). For all the constructs, a shift was observed upon addition of protein, indicating that the constructs can bind DNA. Shown are representative examples of n=3 independent biological replicates. We note that for Sox19b we tested the DBD that was predicted by UniProt and that we used in our study (68aa) as well as a longer version (79aa) that contains additional amino acids that have been shown to be important for DNA binding and interaction with other TFs (2, 3).

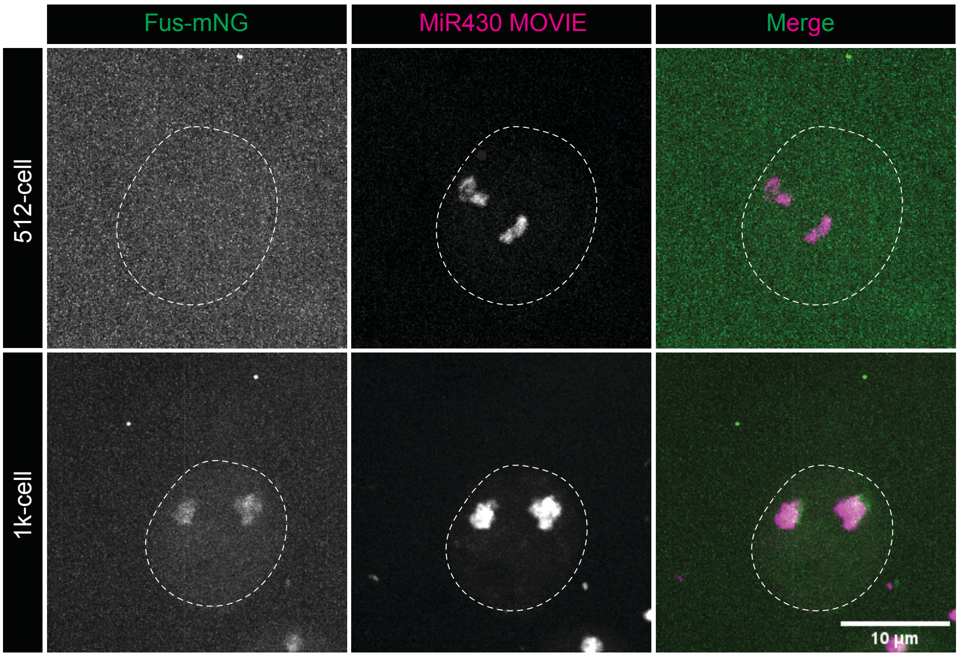

Figure S6. Fus-mNG does not form clusters at 512-cell stage. Visualisation of Fus-mNG and MiR430 transcripts in WT embryos at 512- and 1k-cell stage. At 512-cell stage, no Fus clusters can be detected. At 1k-cell stage, two Fus clusters can be detected. These colocalize with MiR430 transcripts. Shown are representative examples of individual nuclei extracted from spinning disk confocal microscopy during interphase. With N as the number of embryos, and n as the total number of nuclei N ≥5 and n ≥24.

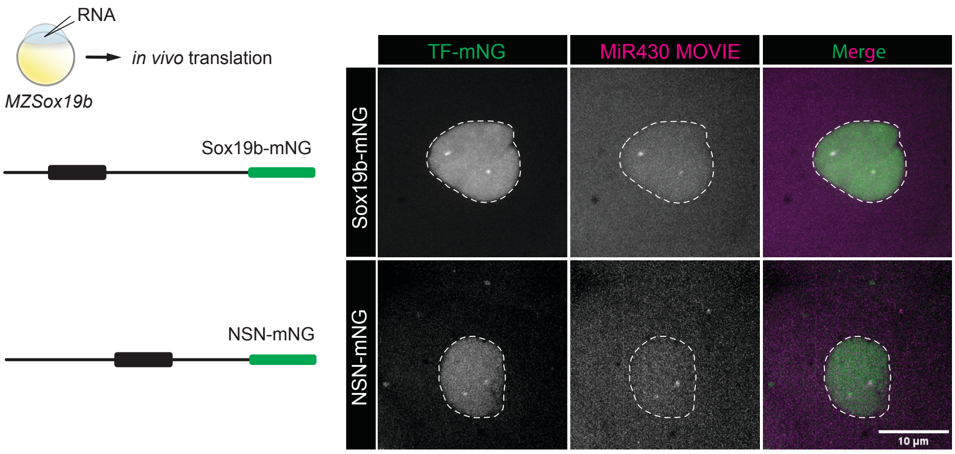

Figure S7. **Specificity in clustering is mediated by the DBD (related to Figure 4).** MZ*sox19b* mutant embryos were injected with FL Sox19b-mNG, or NSN-mNG, in both cases together with MiR430 MOVIE. NSN refers to the chimaeric protein as described in the text. Both proteins form two clusters and these colocalize with a marker for the *mir430* transcription bodies, MiR430 MOVIE. Shown are representative images of individual nuclei extracted from spinning disk confocal microscopy at 512-cell stage during interphase. Images were taken right after mitosis because Sox19b-mNG and NSN-mNG clusters only from transiently. With N as the number of embryos, and n as the number of nuclei N ≥6 and n ≥20.

REFERENCES TO SUPPLEMENTARY FIGURES

1. Dass,R., Mulder,F.A.A. and Nielsen,J.T. (2020) ODiNPred: comprehensive prediction of protein order and disorder. *Scientific Reports 2020 10:1*, **10**, 1–16.
2. Reményi,A., Lins,K., Nissen,L.J., Reinbold,R., Schöler,H.R. and Wilmanns,M. (2003) Crystal structure of a POU/HMG/DNA ternary complex suggests differential assembly of Oct4 and Sox2 on two enhancers. *Genes Dev*, **17**, 2048–2059.
3. Hou,L., Srivastava,Y. and Jauch,R. (2017) Molecular basis for the genome engagement by Sox proteins. *Semin Cell Dev Biol*, **63**, 2–12.
